# Enhanced recovery after surgery in congenital duodenal obstruction

**DOI:** 10.1186/s12876-023-03057-y

**Published:** 2023-11-30

**Authors:** Li-Bo Zhu, Yang-Fang Li, Jun-Tao Shu, Min Xi, Qiang Bai, Jian-Hong Yan, Ling Liu, Cui-Lian Li

**Affiliations:** Department of Neonatal, Kunming Children’s Hospital, No288.Qian Xing Road, Kunming, Yunnan, 650228 China

**Keywords:** Enhanced recovery, Infant, Neonate, Upper gastrointestinal obstruction, Surgery

## Abstract

**Background:**

The present study aims to explore the clinical application of enhanced recovery after surgery (ERAS) in pediatric patients with congenital upper gastrointestinal obstruction (CUGIO).

**Methods:**

A total of 82 pediatric patients with CUGIO admitted to the neonatal intensive care unit in Kunming Children’s Hospital between June 2017 and June 2021 were enrolled in the present study and divided into two groups: the ERAS group (n = 46) and the control group (n = 36). The ERAS management mode was adopted in the ERAS group, and the conventional perioperative management mode was adopted in the control group.

**Results:**

In the ERAS group and the control group, the time to the first postoperative bowel movement was 49.2 ± 16.6 h and 58.4 ± 18.8 h, respectively, and the time to the first postoperative feeding was 79 ± 7.1 h and 125.2 ± 8.3 h, respectively. The differences in the above two indicators between the two groups were statistically significant (P < 0.05). In the ERAS group, the days of parenteral nutrition and the length of hospital stay were 14.5 ± 2.3 d and 18.8 ± 6.4 d, respectively. In the control group, 17.6 ± 2.2 d and 23.1 ± 8.1 d, respectively. The differences in these two indicators between the two groups were statistically significant (P < 0.05).

**Conclusion:**

The ERAS management model had a positive effect on early postoperative recovery in pediatric patients with CUGIO.

## Background

Intestinal atresia is a common congenital gastrointestinal malformation occurring in newborns; the most common causes of upper gastrointestinal obstruction in the neonatal period are duodenal atresia and annular pancreas. Surgery is currently the only effective treatment, and the early recovery of the pediatric patient after surgery is closely correlated with perioperative management.

Enhanced recovery after surgery (ERAS) refers to the application of various perioperative methods with evidence-based effectiveness to reduce surgical stress and the incidence of complications as well as promote rapid postoperative recovery [1]. To date, ERAS has been successfully adopted in adult colorectal resection, orthopedics, gynecology, and urology; however, application in children, especially in the field of neonates, is rarely reported.

An ERAS unit was established in the neonatal intensive care unit (NICU) in Kunming Children’s Hospital, where the authors of the present study worked, in January 2017 with the participation of the department of neonatal surgery, department of anesthesiology, and department of clinical nutrition. Here, the ERAS model was applied to perioperative management of patients with congenital gastrointestinal malformations.

In the present study, the ERAS mode and conventional management were applied to 82 pediatric patients with congenital upper gastrointestinal obstruction (CUGIO). Compared with conventional management, the ERAS mode can indeed accelerate the postoperative rehabilitation of children, which will provide a new mode for the perioperative management of children with congenital duodenal obstruction, and also provide a clinical basis for the application of eras in the field of newborns.

## Materials and methods

### Study subjects

A total of 82 pediatric patients diagnosed with CUGIO who were admitted to the NICU in Kunming Children’s Hospital between June 2017 and June 2021 were enrolled in the present study and randomly divided into two groups by using random number table method and online random software: the ERAS group and the control group.

Inclusion criteria: (1) Patients who met the diagnostic criteria for congenital intestinal atresia in the fifth edition of Practical Neonatology [2] were included in the study; (2) Patients whose mothers had a history of excess amniotic fluid during pregnancy; (3) patients who vomited (mostly bile) shortly after birth or at the first feeding; and (4) patients with abdominal distension, with a main manifestation of epigastric fullness; a double bubble sign might be visible in the standing abdominal radiograph as well as dilated proximal duodenum in gastrointestinal radiography.

Exclusion criteria: (1) patients with low intestinal atresia; (2) patients with multiple organ developmental malformations, congenital chromosomal disorders, and genetic metabolic diseases; (3) neonates with a severe infectious disease; (4) patients with severe neonatal necrotizing enterocolitis and perforation; (5) patients with multiple organ dysfunction or failure; and (6) patients with incomplete information due to various reasons.

The present project was approved by the ethics committee of Kunming Children’s Hospital (Kunming Children’s Hospital ethics number: 20,190,211,005), and the parents of the children voluntarily signed the study-related informed consent form after fully understanding the specifics of the present study.

### Methods

Pediatric patients who met the inclusion criteria were grouped according to the implementation of the ERAS management model and the conventional perioperative management model, respectively. The specific measures of different management models are shown in Table 1. A heated bed was used during surgery to maintain the child’s core temperature at above 36 °C. The intraoperative fluid infusion was heated to 36–37 °C.

**Table 1:** The specific measures of the different management models

Measure	ERAS group (n = 46)	Control group (n = 36)
Pre-operatively		
Publicity and education	Parents were informed of the implementation plan and goals of the ERAS model, and trained to participate in the postoperative care	Traditional health education was conducted by the nurses in the NICU ward to the parents concerning the condition, surgery, and care in the pediatric patient.
**Intra-operatively**		
Mode of surgery	**Laparoscopic exploration was conducted, and the malformed bowel segment was dragged out via the umbilicus with the conduction of the laparoscopic external anastomosis.**	**Laparoscopic exploration was conducted, and the malformed bowel segment was dragged out via the umbilicus with the conduction of the laparoscopic external anastomosis.**
Body temperature management	A variety of insulation measures such as pre-heating, increasing the temperature in the operating room, using a thermoregulator, and **a heated bed were adopted to ensure that the core temperature of the pediatric patient during surgery was > 36 °C.**	**The pediatric patient was warmed with the heated bed to ensure that the core temperature was > 36 °C during surgery.**
Liquid management	**The warmed isotonic compound electrolyte solution was infused at a controlled rate of 6–8 ml/(kg. h).**	**The normothermic isotonic compounded electrolyte solution was infused at a rate of 6–8 ml/(kg. h).**
**Post-operatively**		
Analgesia	**Ultrasound-guided nerve block anesthesia was conducted** with non-nutritive sucking of a soother for analgesia.	**Ultrasound-guided nerve block anesthesia was conducted.**
Early-stage activities	After the condition stabilized (withdrawal of the ventilator and with stable vital signs) in the pediatric patient, the parents in the ERAS management group entered the NICU accompanied by the nursing staff of the management team with preliminary training to complete the basic nursing measures for the pediatric patient, including holding the child, measuring the temperature, feeding, patting the back, and observing the trans-cutaneous oxygen saturation, etc. with a duration of 3–4 h per day.	Postoperatively, the pediatric patient rest in bed and the nurse in the NICU ward completed basic care such as feeding, temperature measurement, and diaper changing.

#### Feeding criteria

When the bowel sounds in the pediatric patient returned to normal with the occurrence of defecation or exhaustion with the drainage volume of postoperative gastrointestinal decompression at < 2 ml/(kg·d), the gastrointestinal drainage tube could be removed with the initiation of the feeding. The deep hydrolyzed protein formula was selected for postoperative feeding. The osmotic pressure of the formula was only 171.8mOsm/l, and the macromolecular milk protein was hydrolyzed into short peptides and amino acids, which was more conducive to the absorption of small intestinal mucosa.

#### Discharge criteria

(1) The vital signs were stable in the pediatric patient (SO2% > 90%, without oxygen inhalation); (2) the amount of total enteral nutrition milk reached 100 ~ 120 ml/(kg·d), with good tolerance after feeding and without vomiting and abdominal distension; (3) the patient had normal spontaneous bowel movements and defecation; and (4) the patient had a normal blood routine test and abdominal X-ray.

#### Observation indicators

Time to the first postoperative bowel movement, time to the first postoperative feeding, length of hospital stay, days of parenteral nutrition, and postoperative complications.

### Statistical methods

The SPSS 20.0 software was adopted for data analysis. The measurement data that satisfied the normal distribution were expressed as means ± standard deviation (x ± s), and the countable data were expressed as percentage (%). The independent samples t-test, analysis of variance, and χ² test was adopted for the comparison between the two groups. A P value of < 0.05 was considered statistically significant.

## Results

### Comparison of general characteristics

A total of 82 pediatric patients who met the inclusion criteria were randomly divided into two groups: the ERAS group (n = 46) and the control group (n = 36). The same group of surgeons participating in the ERAS model operated on patients in both groups. The procedure was conducted via laparoscopic exploration, in which the malformed bowel segment was dragged out through the umbilicus and laparoscopic external anastomosis. The surgical method of duodenal atresia was diaphragm resection duodenal anastomosis, and the surgical method of annular pancreas was duodenal duodenal rhombic anastomosis. The differences in general characteristics (e.g., age, gestational age, birth weight, age at surgery, and type of obstruction) between the two groups were not statistically significant (P > 0.05), and the data were comparable (Table 2).

**Table 2:** Comparison of the general characteristics between the two groups of pediatric patients

		ERAS group	Control group	P value
Type of obstruction	Duodenal atresia	28	22	
	Annular pancreas	18	14	
Age (d)		2.9 ± 1.9	2.8 ± 2.2	0.764
Gestational age (week)		36.1 ± 2.8	36.4 ± 2.7	0.885
Birth weight (g)		2467 ± 396.8	2498 ± 429.6	0.510
Age at operation (d)		3.9 ± 2.1	4.2 ± 2.2	0.816

### Comparison of observation indicators between the two groups

When comparing the evaluation indicators between the two groups, the results suggested that the time to the first postoperative bowel movement, time to the first postoperative feeding, length of hospital stay, and days of parenteral nutrition were all shorter in the ERAS group than in the control group; the differences were statistically significant (P < 0.05; Table 3). The time of operative intervention was longer in the ERAS group then in the control group (P < 0.05; Table 3). Patients in both groups participated in a follow up for 8–12 weeks after discharge. One case of adhesive intestinal obstruction occurred in the ERAS group, and no postoperative complications, such as incisional infection or anastomotic fistula, occurred in either group.

**Table 3:** Comparison of the observation indicators between the two groups of pediatric patients

	ERAS group	Control group	t value	P value
Number of cases	46	36		
The time to first postoperative bowel movement(h)	49.2 ± 16.6	58.4 ± 18.8	2.879	0.018
The time to first postoperative breastfeeding(h)	79 ± 7.1	125.2 ± 8.3	2.567	0.012
The days of parenteral nutrition(d)	14.5 ± 2.3	17.6 ± 2.2	2.281	0.025
Length of hospital stay (d)	18.8 ± 6.4	23.1 ± 8.1	2.264	0.026
Postoperative complication	1	0(0)	0	0
Time of operative intervention (minutes)	54.87 ± 7.14	40.40 ± 5.73	6.120	0.000

## Discussion

ERAS is a multimodal, integrated perioperative pathway aiming to promote patient recovery by reducing stress and the incidence of postoperative complications; this is achieved through evidence-based interventions during the perioperative period [3]. Various evidence-based optimization measures have achieved good results in the field of adult surgery [4]; however, their implementation among pediatric patients, especially in the field of neonatal surgery, is still in the exploratory stage [5].

The expert consensus proposed in the *American Journal of Pediatric Surgery* (2018) suggests 22 ERAS optimization measures that can be applied in the treatment of pediatric patients. The key points are as follows: (1) full pre-operative communication as well as seeking the patient’s understanding and cooperation; (2) no dwelling of the gastric tube and bowel preparation as usual; (3) no need for pre-operative fasting; (4) recommendation of minimally invasive surgery and controlled infusions; (5) conduction of reasonable anesthesia and postoperative analgesia; (6) intraoperative warming; and (7) encouraging of patients to ambulate early and eat early after surgery etc. [6].

Despite the lack of effective evidence-based medical support for these measures, certain data from relevant studies show the efficacy of ERAS in the perioperative management of congenital gastrointestinal anomalies, including jejunal atresia, congenital duodenal obstruction [7], neonatal intestinal malrotation, and congenital heart disease [8–10].

Due to the specificity of the age and disease spectrum in newborns diagnosed with CUGIO included in the present study, the following measures were selectively adopted in the implementation of the ERAS model.

(1) Preoperative publicity and education: compared with ERAS education for adults, ERAS education for newborns was primarily about gaining the understanding and cooperation of the parents. It has been shown that the cooperation and involvement of parents in the implementation of ERAS in children is important for speeding up the postoperative recovery of the pediatric patient [11, 12].

In the present study, an ERAS management team comprising three physicians and three NICU charge nurses (all nurses had at least three years of NICU experience) was established by the project team. Before the operation, members of the management team communicated fully with the parents of the patient in the ERAS group. They were informed about the implementation process and goals of the ERAS management model; they were also provided with training on basic non-medical care measures for newborns as well as knowledge of infection control (e.g., holding the child, changing diapers, feeding, patting the child’s back, monitoring vital signs, and adhering to standard hand hygiene).

The involvement of parents in the ERAS management model increased trust and satisfaction with health care providers compared with conventional preoperative health education.

(2) Intraoperative temperature management: In the ERAS group, various insulation measures (e.g., pre-warming, increasing the temperature in the operating room, and using a thermoregulator and a heated bed) were conducted, along with warming the intraoperative fluid infusion (36–37 °C) to ensure that the intraoperative core temperature of the pediatric patient was > 36 °C.

These measures helped ensure the function of vital organs and the stability of the internal environment; it also directly affected the surgical process and anesthesia resuscitation.

(3) Postoperative pain management: Ultrasound-guided nerve block anesthesia was conducted in both groups for postoperative analgesia. A soother was given to the patients in the ERAS group for non-nutritive sucking during the procedure; this relieved irritability and made the patients feel secure. Use of a soother can reduce the gastrointestinal distension and energy consumption caused by crying, which is conducive to promoting the recovery of gastrointestinal function and reducing the administration of analgesics.

(4) Early postoperative activity: Early postoperative activity facilitates gastrointestinal motility and neurotransmitter secretion, promoting the recovery of gastrointestinal function. Early postoperative ambulation is encouraged in both adult and pediatric patients; newborns are limited to passive early postoperative mobility.

Due to the requirements of ward management and infection control, NICUs in China are basically a closed management model at present. The conventional perioperative management model directs the pediatric patient to rest in bed, with the NICU nurses completing basic care (e.g., feeding, temperature measurement, and diaper changing).

According to the results of several domestic and international studies on family integrated care [13], the involvement of parents in pediatric patient care is important for the health and prognosis of pediatric patients in the NICU; it can promote the recovery of postoperative gastrointestinal function, build a harmonious doctor–patient relationship, and increase the ability of parents to care for their children after discharge.

Therefore, the parents of pediatric patients who participated in the ERAS management model were respectively included as one of the project team members. After training and guidance by the project management team members, the parents entered the NICU after the children were withdrawn from the ventilator with stable vital signs and participated in certain non-medical basic care measures (e.g., holding the child, changing diapers, feeding, and patting the child’s back), with a duration of 3–4 h each time until the pediatric patients were discharged.

The results of the present study revealed that the time to the first postoperative bowel movement, time to the first postoperative feeding, length of hospital stay, and days of parenteral nutrition were all shorter in the ERAS group than in the control group. Therefore, it was suggested that the involvement of parents had a positive effect on the postoperative recovery of pediatric patients with CUGIO.

There were some limitations in the present study. Due to the small sample size included in this study and the fact that only some of the measures in ERAS were implemented in the research process, there is still a lot of work to be done to safely and effectively apply the measures in the ERAS management model to the field of newborns, and more research data is needed to provide evidence-based medical support.

## Conclusion

In summary, certain measures in the ERAS management model might be feasible and effective in promoting early postoperative recovery in pediatric patients with CUGIO when compared with the conventional perioperative management model. The potential reasons for the superiority of ERAS over conventional management in pediatric patients with CUGIO included increased trust and satisfaction with health care providers, improved function of vital organs and internal stability, promotion of gastrointestinal recovery, reduced administration of analgesics, and the involvement of parents in pediatric patient care.

## Data Availability

Data related to the current study are available from the corresponding author on reasonable request.

## Abbreviations

ERAS: enhanced recovery after surgery
CUGIO: congenital upper gastrointestinal obstruction
NICU: neonatal intensive care unit
